# Pridopidine for the Improvement of Motor Function in Patients With Huntington's Disease: A Systematic Review and Meta-Analysis of Randomized Controlled Trials

**DOI:** 10.3389/fneur.2021.658123

**Published:** 2021-05-13

**Authors:** Shujun Chen, Tianyu Liang, Tao Xue, Shouru Xue, Qun Xue

**Affiliations:** ^1^Department of Neurology, The First Affiliated Hospital of Soochow University, Suzhou, China; ^2^Department of Neurosurgery and Brain and Nerve Research Laboratory, The First Affiliated Hospital of Soochow University, Suzhou, China

**Keywords:** pridopidine, Huntington's disease, TMS, MMS, meta-analysis

## Abstract

**Background:** Huntington's disease (HD) is a progressive neurodegenerative disorder. Generally, it is characterized by deficits in cognition, behavior, and movement. Recent studies have shown that pridopidine is a potential and effective drug candidate for the treatment of HD. In the present study, we performed a meta-analysis to evaluate the efficacy and safety of pridopidine in HD.

**Methods:** The MEDLINE, EMBASE, CENTRAL, and Clinicaltrials.gov databases were searched for randomized controlled trials (RCTs) which had that evaluated pridopidine therapy in HD patients.

**Results:** We pooled data from 1,119 patients across four RCTs. Patients in the pridopidine group had a significantly lower Unified Huntington's Disease Rating Scale (UHDRS)-modified Motor Score (mMS) (MD −0.79, 95% CI = −1.46 to −0.11, *p* = 0.02) than those in the placebo group. Additionally, no differences were observed in the UHDRS-Total Motor Score (TMS) (MD −0.91. 95% CI = −2.03 to 0.21, *p* = 0.11) or adverse events (RR 1.06, 95% CI = 0.96 to 1.16, *p* = 0.24) in the pridopidine and placebo groups. In the subgroup analysis, the short-term (≤12 weeks) and long-term (>12 weeks) subgroups exhibited similar efficacy and safety with no statistical significance in TMS, mMS, or adverse events. However, TMS (MD −1.50, 95% CI = −2.87 to −0.12, *p* = 0.03) and mMS (MD −1.03, 95% CI = −1.87 to −0.19, *p* = 0.02) were observed to be improved significantly when the dosage of pridopidine ≥90 mg/day. Additionally, pridopidine (≥90 mg/day) increased total adverse events (RR 1.11, 95% CI = 1.00 to 1.22, *p* = 0.04) compared with placebo. On this basis, we analyzed the incidence of various adverse events when the dosage was ≥90 mg/day. Nonetheless, these results were within the acceptable threshold, although patients developed symptoms, such as nasopharyngitis and insomnia.

**Conclusion:** Pridopidine improved mMS and had no statistical significance in association with TMS or adverse events. Pridopidine (≥90 mg/day) improved TMS and mMS but increased adverse events, such as nasopharyngitis and insomnia. More RCTs were expected to assess pridopidine in HD.

## Introduction

Huntington's disease (HD) is a rare autosomal dominant genetic disease that typically has a mid-life onset and is characterized by chorea, dystonia, incoordination, cognitive decline, and behavioral difficulties, ending in death (1–3). HD is progressive in nature, and once it is acquired, its impact on patients and their families is devastating (4). With the development of modern medical research, it is clear that HD is caused by autosomal dominant CAG trinucleotide repeat amplification of the Huntington protein (HTT) gene on chromosome 4 (5, 6). This ultimately leads to the production of a mutant Huntingtin protein (mHTT) with an abnormally long polyglutamine repeat (7). In terms of etiology, the prevalence of HD is closely related to race. For example, the prevalence of HD is 10.6–13.7 per 100,000 people, and it is rare among people of non-European descent (8). Recently, considerable progress has been made in HD research, and although there are no treatment methods available to prevent its onset or progression, there are a number of potential therapies in development (9, 10). Due to impacts on daily functioning, interference with social activities, gait instability, and threats to personal safety, chorea is undoubtedly a huge obstacle for both the patients themselves and their families (11, 12). The most frequently prescribed drugs for chorea in HD include tetrabenazine, olanzapine, risperidon, tiapride, quetiapine, and aripiprazole (13–15). Among these drugs, tetrabenazine (TBZ), which is an inhibitor of presynaptic vesicular monoamine transporter type 2 (VMAT2) along with a synaptic vesicular amine transport inhibitor and provides sustained reductions in dopaminergic neurotransmission, is the only drug specifically licensed by the US FDA to treat chorea (4). However, TBZ has a relatively short half-life and serious side effects to include depressive symptoms and suicidal behavior (11, 16). In recent years, an increasing number of studies have begun to focus on dopamine antagonists, which block postsynaptic dopamine D2 receptors to suppress chorea (17).

Pridopidine belongs to the new class of D2 receptor antagonists and is also called a dopaminergic stabilizer (18). Pridopidine increases striatal dopaminergic transmission when the dopaminergic tone is low and inhibits the stimulatory actions of dopamine when dopaminergic activity is high (19). Pridopidine can reverse and improve behavioral states in a concentration-dependent manner without having major effects on normal mental activity (20, 21). During the past two decades, researchers have been interested in pridopidine because of its unique pharmacology (22). The efficacy and safety of pridopidine remain to be revealed. Among the randomized controlled trials (RCTs) conducted to determine the benefits of using pridopidine in patients with HD, varying conclusions have been drawn. The HART, MermaiHD, and Lundin2010 results suggest that pridopidine has a trend toward a positive effect on the UHDRS-total motor score (UHDRS-TMS) and modification of the motor score (mMS), indicating that it may improve motor function in HD (23–25). However, PRIDE-HD reported that pridopidine did not significantly differ from placebo (26). All studies of pridopidine in HD have shown good safety and tolerability (23–26). These studies have led us to comprehensively reconsider the role of pridopidine and its clinical application.

Based on these findings, we found that the effectiveness and safety of pridopidine have not been systematically evaluated in prospective series or RCTs. Therefore, we conducted a meta-analysis of pooled data from previous clinical trials to investigate the value of pridopidine and to explore the potential factors that might influence the efficacy and safety of pridopidine.

## Methods

### Study Protocol

In accordance with the Cochrane Collaboration format, we initially drew up a research protocol (27).

### Eligibility Criteria

The inclusion criteria were based on the following points: (a) Study type: RCT; (b) Language limitation: English only; (c) Participants: patients with HD; (d) Intervention: Pridopidine or placebo; (e) Outcomes: TMS, mMS and adverse events. The exclusion criteria were as follows: (a) Study type: cohort studies, case reports, case reviews, and retrospective studies; (b) Control: positive control.

### Information Sources and Search Strategy

Two independent authors (SJC and TYL) systematically searched four main databases: MEDLINE, EMBASE, CENTRAL, and clinicaltrials.gov. The search strategy combined “Pridopidine” AND “Huntington's Disease” for MEDLINE. The search strategy for EMBASE, CENTRAL, and clinicaltrials.gov was similar to that used for MEDLINE. Studies that matched the abstracts and titles were searched.

### Study Selection and Data Collection

All results obtained from the reference lists of RCTs and electronic databases were appraised by two independent investigators (SJC and TYL) based on the eligibility criteria previously mentioned. After stringent screening and assessment, all essential information was extracted from the RCTs (Table 1 and Supplementary Table 1).

**Table 1:** Characteristics of the Included Studies.

**Study**	**Countries**	**Centers**	**Publications**	**Phase**	**Durations (weeks)**	**Treatment group**, **(No. of participants)**	**Age range**	**Mean age ± SD (year)**		**Female (%)**		**Funder**
								**Pridopidine**	**placebo**	**Pridopidine**	**placebo**	
Reilmann et al. (26) (NCT02006472)	12	53	Lancet Neurology	II	26, 52	Placebo (82) vs. Pridopidine 45 mg/d (81) vs. Pridopidine 67.5 mg/d (82) vs. Pridopidine 90 mg/d (81) vs. Pridopidine 112.5 mg/d (82)	≥21 y	50.4 ± 12.0	50.3 ± 11.3	50	49	Pharma
HART et al. (23) (NCT00724048)	2	27	Movement Disorders	II	12	Placebo (58) vs. Pridopidine 20 mg/d (56) vs. Pridopidine 45 mg/d (55) vs. Pridopidine 90 mg/d (58)	≥30 y	51.9 ± 10.5	50.4 ± 10.5	52.1	56.9	Pharma
de Yebenes et al. (25) (NCT00665223)	8	32	Lancet Neurology	III	26	Placebo (144) vs. Pridopidine 45 mg/d (148) vs. Pridopidine 90 mg/d (145)	≥30 y	51.4 ± 10.9	49.1 ± 9.6	49.8	53	Pharma
Lundin et al. (24)	2	6	Clinical Neuropharmacology	I/II	4	Placebo (30) vs. Pridopidine 50 mg/d (28)	25 y-75y	50.2 ± 7	56 ± 10	29	37	Pharma

*PRIDE-HD, Pridopidine Dose Evaluation in Huntington's Disease; HART, Huntington's Disease ACR16 Randomized Trial; MermaiHD, Multinational European Multicentre ACR16 Study in Huntington's Disease*.

### Risk of Bias

The risk of bias in individual studies was assessed based on Review Manager 5.3 software. The consolidated criteria of the Cochrane collaboration were used to assess biases, including selection bias, performance bias, detection bias, attrition bias, reporting bias, and other potential biases.

### Summary Measures and Synthesis of Results

All data were assessed by two investigators (SJC and TYL) using Review Manager (Version 5.3) software. Dichotomous outcomes were analyzed as the risk ratio [relative risk (RR); 95% confidence interval (CI)]. All analyses were conducted using a fixed-effects model. The *I*^2^ and *p*-values were applied to evaluate the heterogeneity among the included studies. When *I*^2^ > 50%, we assumed that the data were heterogeneous. When *I*^2^ < 30%, we assumed that the data showed mild heterogeneity, and moderate heterogeneity was defined as 30% < *I*^2^ < 50%. In addition, we performed subgroup analyses according to different treatment endpoints and dosages of pridopidine. A two-tailed test was conducted, and a *p*-value <0.05 was regarded as significant for all analyses.

## Results

**Figure 1:**
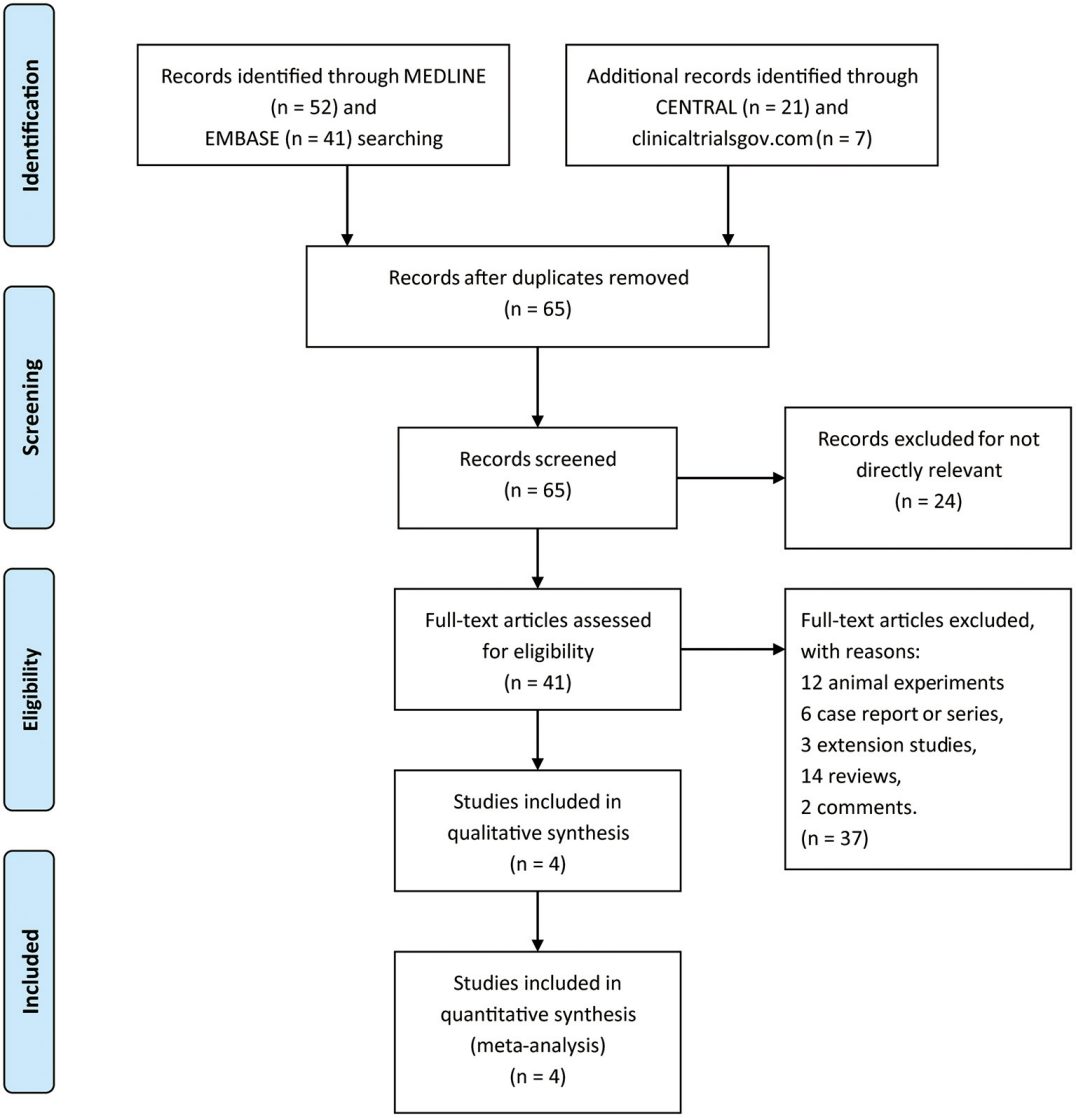
The study search, selection, and inclusion process.

A total of 121 titles and abstracts were identified through MEDLINE, EMBASE, CENTRAL, and clinicaltrials.gov (Figure 1). After eliminating duplicates and unrelated records, 41 full-text articles were appraised for eligibility. In addition, 37 articles were excluded due to the restriction of publication types: 12 animal experiments, 6 case reports or series, 3 extension studies, 14 reviews, and 2 comments. Ultimately, four RCTs containing 1,119 patients (pridopidine, *n* = 806; placebo, *n* = 313) were included in the qualitative and quantitative synthesis (Figure 1). The main characteristics of the included studies are listed in Table 1.

### Outcomes Analysis

All four RCTs (23–26) enrolling 1,119 patients were pooled for the analysis of outcomes from two aspects: efficacy and safety. In this meta-analysis, the primary efficacy endpoints were evaluated as the change in the Unified Huntington's Disease Rating Scale-Total Motor Score (UHDRS-TMS) and the modified Motor Score (mMS) from baseline to after pridopidine or placebo therapy.

**Figure 2:**
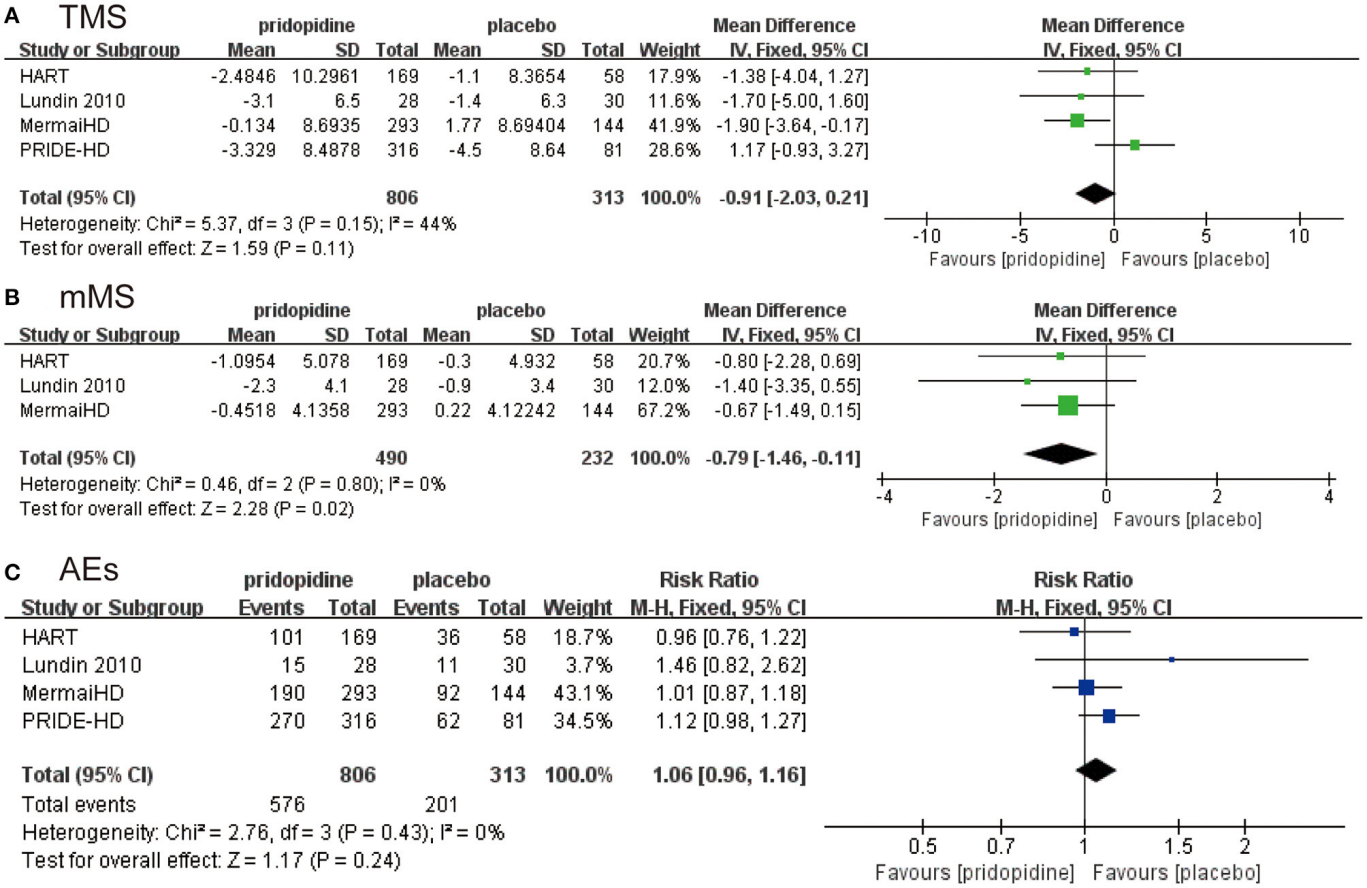
The pooled relative risk of efficacy outcome. The blue diamond indicates the estimated risk ratio (95% confidence interval), and the green diamond indicates the mean difference (95% confidence interval) for all patients together. **(A)** TMS. **(B)** mMS. **(C)** adverse events.

As shown in Figure 2A, there was no significant difference in the reduction of TMS between the pridopidine and the placebo groups (MD −0.91, 95% CI = −2.03 to 0.21, *p* = 0.11). The heterogeneity of TMS was 44.0%, with a *p*-value for heterogeneity of 0.11. To determine the source of heterogeneity, sensitivity analysis was carried out, and the results showed that all the consolidated results were stable (Supplementary Figure 1). In addition, patients in the pridopidine group exhibited significant improvement in mMS (MD −0.79, 95% CI = −1.46 to −0.11, *p* = 0.02; Figure 2B) compared with those in the placebo group. The safety outcomes were assessed by adverse events. The frequencies of adverse events (RR 1.06, 95% CI = 0.96 to 1.16, *p* = 0.24; Figure 2C) showed no significant differences between the pridopidine and the placebo groups.

### Subgroup Analysis

Subgroup analyses were performed to assess the influence of different treatment periods (≤12 weeks/>12 weeks) and different dosages of pridopidine (<90 mg/day/≥90 mg/day).

**Figure 3:**
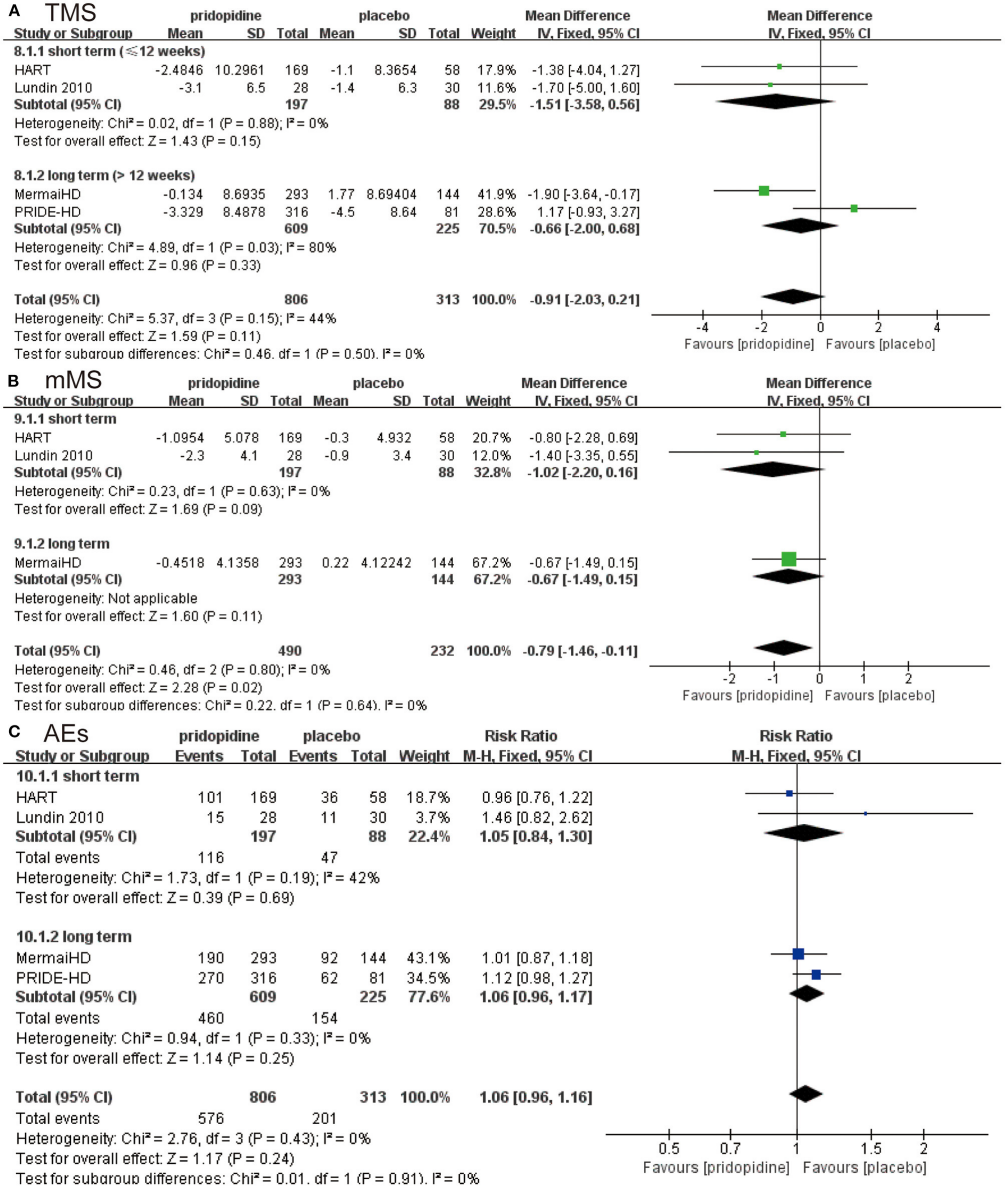
Subgroup analysis of different treatment periods (≤12 weeks/>12 weeks) on efficacy and safety outcomes. The blue diamond indicates the estimated risk ratio (95% confidence interval), and the green diamond indicates the mean difference (95% confidence interval) for all patients together. **(A)** TMS. **(B)** mMS. **(C)** adverse events.

For TMS, mMS, and adverse events, there were no significant differences between pridopidine and placebo in the short term (≤12 weeks) (TMS: MD −1.51, 95% CI = −3.58 to 0.56, *p* = 0.15, pridopidine, *n* = 197, placebo, *n* = 88; mMS: MD −1.02, 95% CI = −2.20 to 0.16, *p* = 0.09, pridopidine, *n* = 197, placebo, *n* = 88; adverse events: RR 1.05, 95% CI = 0.84 to 1.30, *p* = 0.69, pridopidine, *n* = 197, placebo, *n* = 88; Figures 3A-C) or long term (>12 weeks) (TMS: MD −0.66, 95% CI = −2.00 to 0.68, *p* = 0.33, pridopidine, *n* = 609, placebo, *n* = 225; mMS: MD −0.67, 95% CI = −1.49 to 0.15, *p* = 0.11, pridopidine, *n* = 293, placebo, *n* = 144; adverse events: RR 1.06, 95% CI = 0.96 to 1.17, *p* = 0.25, pridopidine, *n* = 609, placebo, *n* = 225; Figures 3A-C) subgroups. The heterogeneity of TMS in the long-term subgroup was 80%, with a *p*-value for heterogeneity of 0.03. Additionally, the heterogeneity of adverse events in the short-term subgroup was 42%, with a *p*-value for heterogeneity of 0.19. Other subgroups showed mild heterogeneity, as shown in Figure 3.

**Figure 4:**
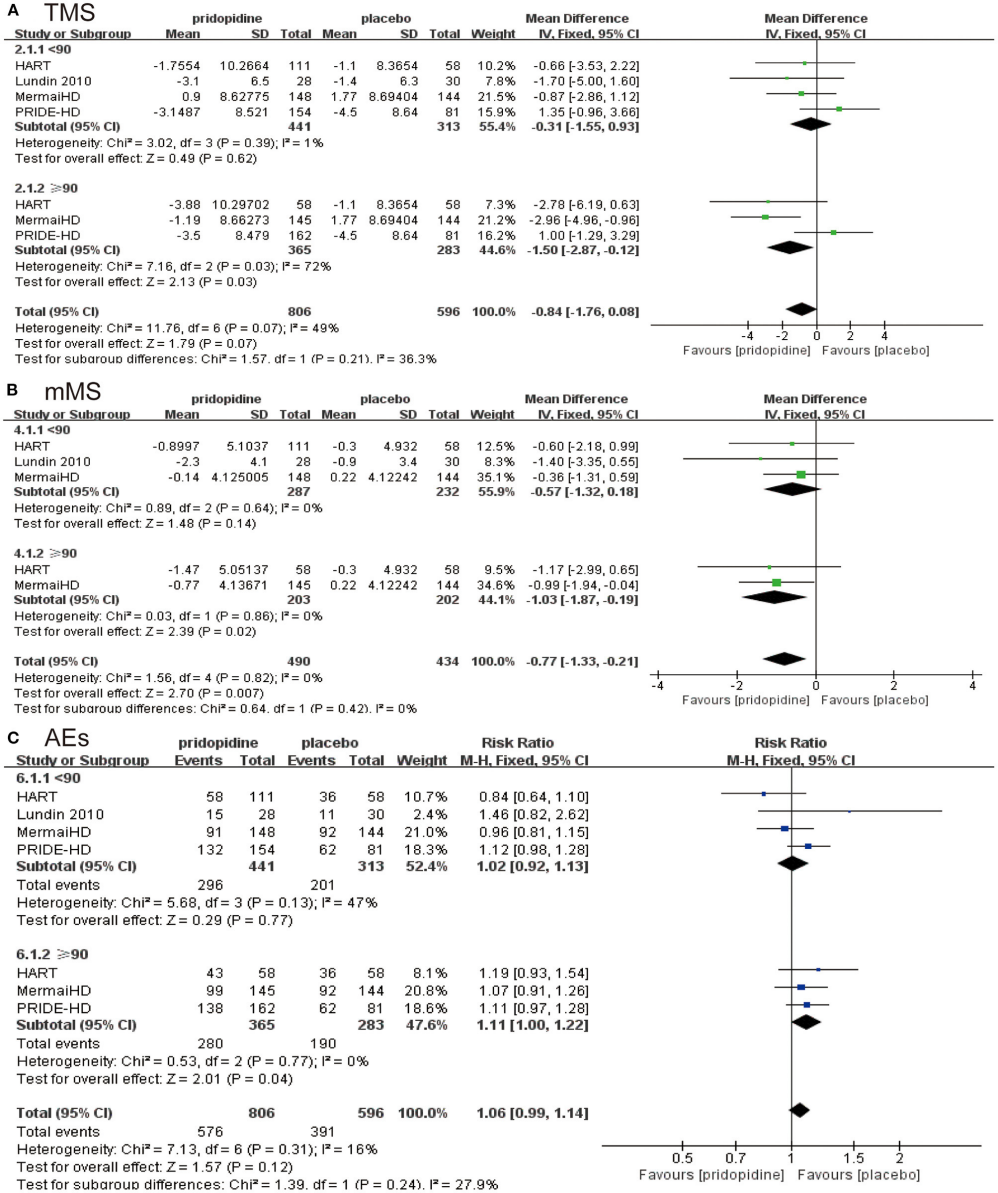
Subgroup analysis of different dosages of pridopidine (<90 mg/day/≥90 mg/day) on efficacy and safety outcomes. The blue diamond indicates the estimated risk ratio (95% confidence interval), and the green diamond indicates the mean difference (95% confidence interval) for all patients together. **(A)** TMS. **(B)** mMS. **(C)** adverse events.

In the lower-dosage subgroup (<90 mg/day), pridopidine had a similar TMS (MD −0.31, 95% CI = −1.55 to 0.93, *p* = 0.62, pridopidine, *n* = 441, placebo, *n* = 313; Figure 4A), mMS (MD −0.57, 95% CI = −1.32 to 0.18, *p* = 0.14, pridopidine, *n* = 287, placebo, *n* = 232; Figure 4B) and adverse events (RR 1.02, 95% CI = 0.92 to 1.13, *p* = 0.77, pridopidine, *n* = 441, placebo, *n* = 313; Figure 4C) as placebo. However, a higher dosage of pridopidine (≥90 mg/day) significantly decreased TMS (MD −1.50, 95% CI = −2.87 to −0.12, *p* = 0.03, pridopidine, *n* = 365, placebo, *n* = 283; Figure 4A) and mMS (MD −1.03, 95% CI = −1.87 to −0.19, *p* = 0.02, pridopidine, *n* = 203, placebo, *n* = 202; Figure 4B) and was associated with more adverse events (RR 1.11, 95% CI = 1.00 to 1.22, *p* = 0.04, pridopidine, *n* = 365, placebo, *n* = 283; Figure 4C) than placebo. The heterogeneity of TMS in the higher-dosage subgroup was 72%, with a *p*-value for heterogeneity of 0.03. Additionally, the heterogeneity of adverse events in the lower-dosage subgroup was 47%, with a *p*-value for heterogeneity of 0.13. Other subgroups showed mild heterogeneity in Figure 4.

We implemented a meta-analysis of adverse effects between pridopidine (≥90 mg/day) and placebo groups. Diarrhea; nausea, vomiting and dry mouth; fatigue; nasopharyngitis; urinary tract infection; fall; headache; dizziness; chorea; depression; and insomnia were the most frequently reported adverse events in the higher dosage of pridopidine (≥90 mg/day). Compared with the placebo group, the higher-dosage pridopidine (≥90 mg/day) groups were more likely to report nasopharyngitis (RR 2.01, 95% CI = 1.10 to 3.69, *p* = 0.02; Table 2) and insomnia (RR 2.23, 95% CI = 1.00 to 4.98, *p* = 0.05; Table 2) and reduced frequency of fatigue (RR 0.51, 95% CI = 0.26 to 1.01, *p* = 0.05; Table 2). The rest of the adverse events had no significant differences between higher dosages of pridopidine and placebo (dry mouth: RR 6.78, 95% CI = 0.86 to 53.33, *p* = 0.07; dizziness: RR 2.06, 95% CI = 0.93 to 4.56, *p* = 0.08; diarrhea: RR 1.29, 95% CI = 0.73 to 2.27, *p* = 0.38; nausea and vomiting: RR 0.91, 95% CI = 0.52 to 1.59, *p* = 0.73; urinary tract infection: RR 1.59, 95% CI = 0.60 to 4.24, *p* = 0.35; fall: RR 1.07, 95% CI = 0.71 to 1.59, *p* = 0.76; headache: RR 1.16, 95% CI = 0.58 to 2.33, *p* = 0.68; chorea: RR 1.59, 95% CI = 0.78 to 3.25, *p* = 0.20; depression: RR 0.69, 95% CI = 0.34 to 1.41, *p* = 0.31; Table 2).

**Table 2:** Meta-analysis of adverse effects between pridopidine (≥90 mg/day) and placebo groups.

	**Pooled estimates Heterogeneity**				
	**RR (95% CI)**	***P*-value**	***X^**2**^***	***P*-value**	***I^**2**^***
**Gastrointestinal disorders**					
Diarrhoea	1.29 [0.73, 2.27]	0.38	0.92	0.63	0%
Nausea and vomiting	0.91 [0.52, 1.59]	0.73	1.26	0.53	0%
Dry mouth	6.78 [0.86, 53.33]	0.07	0.00	0.98	0%
**General disorders**					
Fatigue	0.51 [0.26, 1.01]	0.05	0.15	0.93	0%
**Infections**					
Nasopharyngitis	2.01 [1.10, 3.69]	0.02	0.17	0.92	0%
Urinary tract infection	1.59 [0.60, 4.24]	0.35	1.03	0.31	3%
**Injury**					
Fall	1.07 [0.71, 1.59]	0.76	0.95	0.62	0%
**Nervous system disorders**					
Headache	1.16 [0.58, 2.33]	0.68	0.04	0.84	0%
Dizziness	2.06 [0.93, 4.56]	0.08	2.27	0.32	12%
Chorea	1.59 [0.78, 3.25]	0.20	1.92	0.38	0%
**Psychiatric disorders**					
Depression	0.69 [0.34, 1.41]	0.31	0.19	0.91	0%
Insomnia	2.23 [1.00, 4.98]	0.05	0.63	0.43	0%

*RR, relative risk; CI, confidence interval*.

### Risk of Bias in the Included Studies

**Figure 5:**
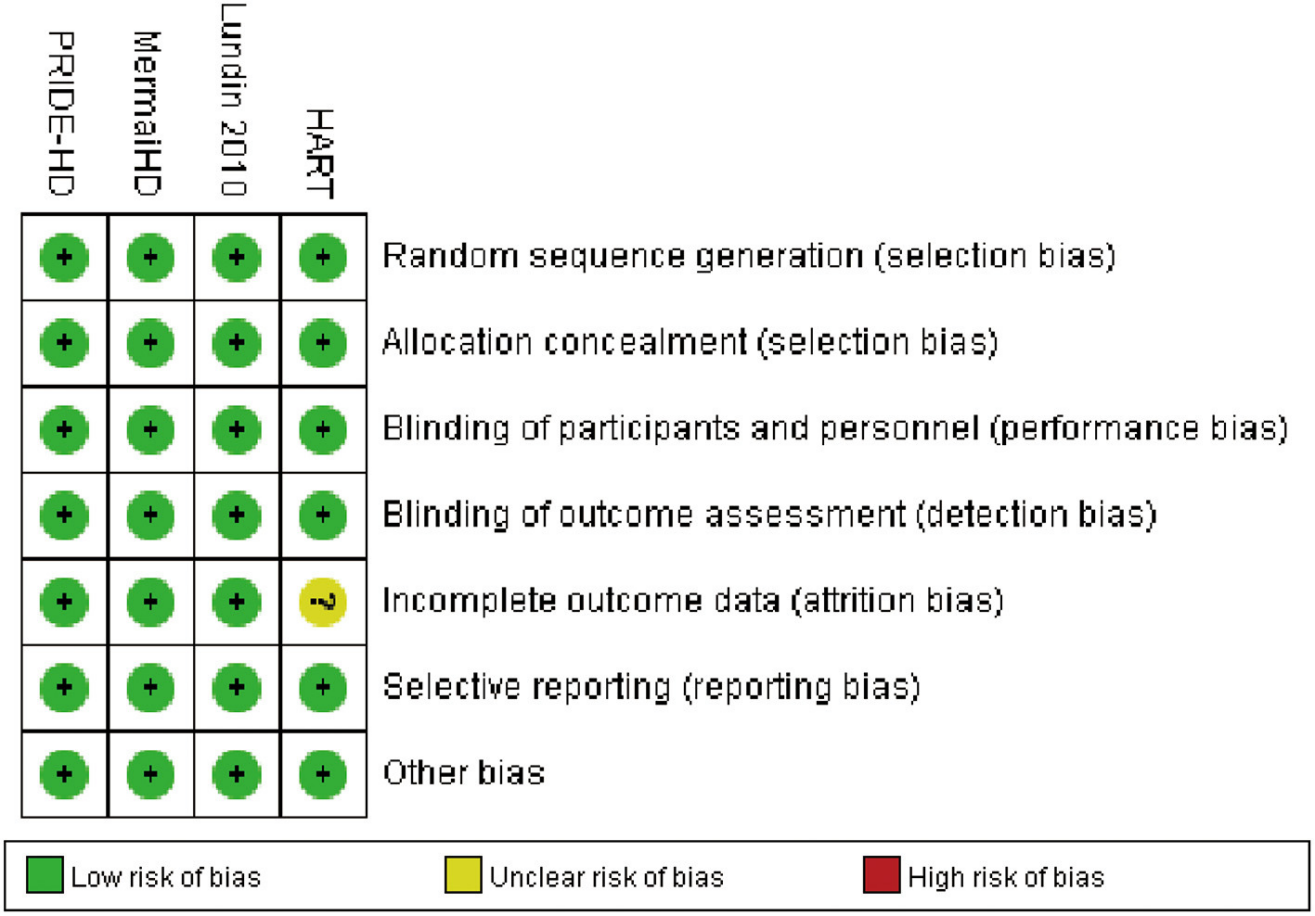
Risk of bias: A summary table for each risk of bias item for each study.

Full details about the risk of bias in the four RCTs are exhibited in Figure 5. For incomplete outcome data, the risk of bias was high in one study (HART 2013) and low in the other three studies. All four included RCTs showed a low risk of selection bias, performance bias, detection bias, reporting bias, and other potential biases. In addition, publication bias could not be assessed by graphical aids and/or statistical tests because there were fewer than 10 included studies.

## Discussion

This meta-analysis showed that pridopidine improved mMS instead of TMS and did not increase adverse events compared with placebo in patients with HD. Therefore, we considered pridopidine to be safe and potentially effective in HD.

In conclusion, four RCTs met our criteria for inclusion (specific information is summarized in Table 1 and Supplementary Table 1). The TMS and mMS from those RCTs were evaluated by researchers. We started by analyzing the evaluation of TMS in these articles, and the results showed that the overall TMS of patients taking pridopidine was not superior to that of the placebo group. Moreover, the heterogeneity was moderate, with *I*^2^ = 44.0% (Figure 2A). As observed in Supplementary Figure 1, we conducted a sensitivity analysis and then found that the data pooled from four trials were stable. Therefore, we did not exclude any trials and concluded that pridopidine did not decrease TMS. However, the result was uncertain due to heterogeneity. In addition, Figure 2B shows that patients in the pridopidine group had significantly lower mMS than those in the placebo group, which indicated that pridopidine improved the symptoms in HD patients in some aspects. Although PRIDE-HD, the largest and most recent study, did not provide the data, mMS as a subset of TMS was the primary outcome of MermaiHD and HART mainly reflecting voluntary movement of patients. This suggested that mMS was an important indicator to evaluate the motor function of HD patients. Therefore, we took it as an efficacy outcome of our study. The result of mMS was not unassailable considering the lack of PRIDE-HD data. Nevertheless, based on the current meta-analysis, we still determined that pridopidine can improve mMS and is ultimately beneficial for HD patients. More clinical trials are expected to reconfirm the abovementioned issues.

The mechanism of pridopidine's treating HD mainly included three aspects (28). Initially, pridopidine, a dopamine D2 receptor (D2R) antagonist that competitively combines with D2R, can attenuate the suppressive effect on DA transport and release vesicles, increase GABA output *via* an indirect pathway and, therefore, relieve involuntary movements (20, 29). Moreover, pridopidine can induce DA release in the frontal cortex and facilitate DA–D1R interactions *via* a direct pathway because most of D2R was occupied by pridopidine. This also increases GABA output and simultaneously improves voluntary movements (20, 30). Second, pridopidine is a sigma-1 receptor (S1R) agonist, and the affinity of pridopidine for S1R is more than 100-fold higher than that for D2R (31, 32). It exerts a neuroprotective effect by increasing BDNF expression and regulating PI3/AKT kinase signaling (33, 34). Another approach was pridopidine interaction with S1R and activation of the SGK1 gene involved in the corticosteroid pathway to reduce neuronal sensitivity to toxic mHtt protein (35, 36). Third, pridopidine acts indirectly on NMDA receptors by increasing the expression of the activity-regulated cytoskeletal protein (Arc) gene in the frontal cortex, thus promoting synaptic NMDAR signaling to play a protective role (37). Although the molecular mechanism of pridopidine acting on HD is well-illustrated, clinical trials are needed to demonstrate the therapeutic efficacy and safety of pridopidine; therefore, we pooled RCTs focusing on pridopidine for the treatment of HD. A meta-analysis was carried out to obtain evidence that pridopidine is effective in the treatment of HD after the data were included.

Generally, in clinical research, the side effects of drugs are considered first when evaluating whether the drug is suitable for the treatment of the related disease. By considering the advantages and disadvantages, researchers must understand the importance of drug safety, which is also important compared with the curative effect. In this work, we compared the incidence of adverse events in the two groups of patients, and the subsequent results yielded convincing outcomes (Figure 2C). In terms of the incidence of adverse events, no significant change was observed in the occurrence of side effects, regardless of whether the drug was taken. This indicated that pridopidine is still a relatively safe drug. Although the use of pridopidine for HD is safe and effective in general, we still need to evaluate the use of pridopidine in a profound way.

Based on the four RCTs, we selected the time and dose of medication for evaluating TMS and mMS. When we examined the duration of the medication, we used 12 weeks as the break point. Specifically, fewer than 12 weeks was considered the short term, and more than 12 weeks was considered the long term. In terms of dose, we separated the included subjects at the turning point of 90 mg, which formed two independent evaluation indicators. From the perspective of medication duration, no significant association was observed between TMS (Figure 3A)/mMS (Figure 3B) and duration of medication in the pridopidine and placebo groups. Our study also showed that the incidence of adverse events was not significantly related to the duration of medication (Figure 3C). This ultimately means that pridopidine can effectively improve the symptoms of HD patients, regardless of the duration of medication; therefore, we cannot only focus on the duration of medication. Interestingly, the dosage of the drug has shown promising results for HD patients. While evaluating the TMS of the two groups, we found that symptom improvement was not significant when the daily dose of pridopidine was <90 mg (Figure 4A). However, when the daily dose was higher than 90 mg, the TMS decreased significantly (Figure 4A). These results suggest that the symptoms of HD patients with daily doses higher than 90 mg were improved significantly. Further investigation showed that the changes in mMS were consistent with TMS (Figure 4B). Similarly, the occurrence of adverse events varied. Two structures suggested that the dose was closely related to the therapeutic effect of the drug. Thus, we can conclude that pridopidine treatment is implemented by adjusting the dosage and the length of time. However, no exact relationship was observed in association with the medication time. We expect that it can provide strong evidence for clinical therapeutics. Although our research has shown that the dosage of pridopidine (≥90 mg/day) can help patients significantly, the scope of security dosage needs a large number of clinical trials for a comprehensive demonstration.

We became very interested in the occurrence of adverse events in the higher-dosage (≥90 mg/day) subgroup and wondered which adverse events had a higher incidence. Next, a meta-analysis of adverse events between pridopidine (≥90 mg/day) and placebo groups was carried out (Table 2). We analyzed each of the specific adverse events at a dosage of ≥90 mg/day. It is worth noting that the obtained results were not consistent. The incidence of two adverse events showed increasing trends (**nasopharyngitis and insomnia)**. Surprisingly, one adverse event (**fatigue**) was reduced in patients who were administered pridopidine at ≥90 mg/day compared to patients in the placebo group. The occurrence of other reported adverse events did not change significantly. Overall, the effects of these two adverse events on patients were relatively mild compared with the symptoms of HD. Therefore, we can conclude that pridopidine is safe and effective for HD treatment.

To the best of our knowledge, the present study is the first meta-analysis to compare pridopidine and placebo using evidence only from RCTs. This was the most appropriate way we could divide risk factors evenly over these two groups. However, the limitations of this meta-analysis were as follows. First, this meta-analysis was conducted based on limited statistics. We only pooled four published RCTs with 1,119 patients to assess the efficacy and safety of pridopidine for HD. Furthermore, this meta-analysis was not registered prior to data collection. In addition, the included RCTs exhibited heterogeneity, which is shown in Figure 2A, for TMS (*I*^2^ = 44%). The sensitivity analysis illustrated that all the consolidated results were stable; however, the disadvantages cannot be ignored.

## Conclusion

In summary, our meta-analysis demonstrated that pridopidine therapy decreased mMS in patients with HD. However, it did not significantly improve TMS. In addition, it also proved that the use of pridopidine was safe. Based on current studies, despite increasing the occurrence of nasopharyngitis and insomnia, a higher dosage of pridopidine (≥90 mg/day) is recommended for HD due to its greater efficacy in improving motor function. Finally, we expect that more RCTs will be implemented in future studies to evaluate pridopidine in HD.

## Data Availability Statement

The raw data supporting the conclusions of this article will be made available by the authors, without undue reservation.

## Author Contributions

SC and TL designed the study, developed the analysis plan, analyzed the data, performed the meta-analysis, and contributed to the writing of the article. QX and TX revised the manuscript and polish the language. All authors contributed to the article and approved the submitted version.

## Conflict of Interest

The authors declare that the research was conducted in the absence of any commercial or financial relationships that could be construed as a potential conflict of interest.
