# Supplementary material for: Pridopidine for the Improvement of Motor Function in Patients With Huntington's Disease: A Systematic Review and Meta-Analysis of Randomized Controlled Trials
Source: Front Neurol. 2021 May 13;12:658123. doi: 10.3389/fneur.2021.658123 (PMC8159155; doi:10.3389/fneur.2021.658123)

Pridopidine for the Improvement of Motor Function in Patients with Huntington's Disease: A Systematic Review and Meta-Analysis of Randomized Controlled Trials

Shujun Chen[1,#],Tianyu Liang[2,#], Tao Xue [2,*],Shouru Xue[1,*],Qun Xue[1,*]

[1] *Department of Neurology, The First Affiliated Hospital of Soochow University, 188 Shizi Street, Suzhou, 215006, Jiangsu Province, China*

[2]*Department of Neurosurgery & Brain and Nerve Research Laboratory, The First Affiliated Hospital of Soochow University, 188 Shizi Street, Suzhou 215006, Jiangsu Province, China*

Complete co-author email addresses:

Shujun Chen, E-mail: 1026443927@qq.com

Tianyu Liang, E-mail:liangtianyu1111@163.com

Tao Xue, E-mail:2992326676@qq.com

Shouru Xue, E-mail:xueshouru@suda.edu.cn

Qun Xue, E-mail: qxue_sz@163.com

[#] Shujun Chen and Tianyu Liang contribute equally to this work.

*Corresponding author: Tao Xue or Shouru Xue or Qun Xue. Department of Neurosurgery & Brain and Nerve Research Laboratory or Department of Neurology, The First Affiliated Hospital of Soochow University, 188 Shizi Street, Suzhou 215006, China. E-mail address: 2992326676@qq.com or xueshouru@suda.edu.cn or qxue_sz@163.com

Table S1 Inclusion, exclusion criteria, and outcome assessments of the included studies

Study	Inclusion Criteria	Exclusion Criteria	Efficacy outcomes	Safety outcomes
PRIDE-HD 2019 (NCT02006472)	Eligible patients were aged 21 years and older and had genetically confirmed presence of 36 or more cytosine-adenine-guanine repeats in one huntingtin gene and symptoms clinically indicative of Huntington's disease, as defined by diagnostic confidence level 4 (i.e., motor abnormalities that are unequivocal signs of Huntington's disease [99% confidence]). Clinical onset after age 18 years was required to avoid recruitment of individuals with juvenile-onset Huntington's disease. Participants had to be clinically	Exclusion criteria were pregnant or lactating women; inadequate contraception (either sex); history of epilepsy or seizures within the past 5 years; use of disallowed neuroleptics, strong cytochrome P450 2D6 inhibitors, or tetrabenazine within 6 weeks of randomization and throughout the study; and a known history or risk of long QT syndrome, prolonged QT corrected for heart rate with Fridericia's correction (QTcF) intervals (>450ms) on electrocardiogram (ECG), or clinically significant heart disease. Patients with previous exposure to pridopidine or any investigational	The primary outcome was change from baseline to week 26 in the TMS of the UHDRS.<br>The secondary outcome was the modified physical performance test at week 26.	adverse events, serious adverse events (defined in the protocol), laboratory tests, vital signs, 12-lead ECG, and the Columbia Suicide Severity Rating Scale.

symptomatic and ambulatory, with screening values of at least 25 points for the UHDRS-TMS and 90% or less for the UHDRS independence score. The availability and willingness of a caregiver or companion (someone attending to the participant at least two to three times per week for at least 3 h each time) to accompany the participant to study visits was mandatory to assess caregiver feedback in secondary endpoints. Participants treated with permitted neuroleptics, anti-depressants, or other psychotropic medications were required to have been on a stable dose for 6 weeks or more before randomization.

product within 6 weeks of screening were also excluded.

HART 2013 (NCT00724048)	Participants were men and women at least 30 years of age with features clinically diagnosed as HD and either a positive family history of HD or a CAG repeats length of 36 or greater. Included subjects were able to provide written consent, take oral medication, comply with study procedures, and travel for study visits; had a caregiver or family member available for select visits; had a modified Motor Score (mMS) ⩾10 points on screening; and were on allowed antidepressants or psychotropic medication at a stable dosage for at least 6 weeks before baseline.	Exclusion criteria included treatment with antipsychotic medications within 8 weeks and tetrabenazine within 12 weeks of the baseline visit. Given reports of seizures in preclinical studies at very high plasma concentrations of pridopidine, medications known to lower the seizure threshold (including antiarrhythmics and antibiotics) were disallowed as a precaution. Other exclusion criteria included history of epilepsy, known seizures of any cause, use of investigational products within 4 weeks of baseline, metoclopramide use within 12 weeks of baseline, history of deep brain stimulation or other surgical procedure aimed at improving HD symptoms, prolonged QT interval on a screening electrocardiogram	The primary outcome variable was the change in voluntary motor function from baseline to week 12, as measured by the mMS.<br><br>Secondary outcome measures were changes from baseline to week 12 in the UHDRS components including the UHDRS-TMS, total UHDRS behavioral frequency and frequency 3 severity scores, UHDRS cognitive scales (Stroop Word Reading, Color Naming, and Interference tests, Symbol Digit Modalities Test, Verbal Fluency), UHDRS functional scales including the Total Functional Capacity score, Independence Scale score, and Functional Checklist score, and UHDRS motor scale sub scores	Safety outcomes were ascertained by asking about adverse events (AEs) at each visit and with each telephone contact, as well as by examining changes in laboratory values (basic chemistries, hematology, liver function tests, urinalysis), vital signs, and ECGs.

		(ECG) or significant cardiac history, creatinine clearance<40 mL/minute at screening, any clinically significant laboratory abnormality at screening, clinically significant hepatic or renal impairment, severe intercurrent illness, alcohol or drug abuse, suicidal ideation, women pregnant or nursing, women intending to become pregnant during the study period, women of childbearing potential not using adequate contraception, and any previous participation in a trial of pridopidine.	(maximal chorea, maximal dystonia, balance/gait[gait, tandem walking, retropulsion pull test], eye movements [ocular pursuit, saccade initiation, saccade velocity], and hand movements [finger taps, pronation/supination, Luria sequence]). The 12-week changes intotal Hospital Anxiety Depression Scale score and the Trail-making A test (expressed as circles per second), as well as the CGI-C at week 12, were also assessed.	
MermaiHD 2011 (NCT00665223)	included patients with Huntington's disease (on the basis of clinical features and the presence of ⩾36 CAG repeats) who were aged 30 years or older (to avoid recruitment of patients with	We excluded patients who were pregnant, lactating, or fertile women not using contraception, patients who received disallowed antipsychotics or tetrabenazine in the 12 weeks before randomization, and patients who	Our primary outcome measure was change in the mMS from baseline to week 26. Our prespecified secondary outcome measures were the clinical global impression	Safety outcomes were adverse events including fall, chorea, diarrhoea, dizziness, nausea, nasopharyngitis,

juvenile disease), were ambulatory, and had a modified motor score (mMS; derived from the unified Huntington's disease rating scale [UHDRS]) of 10 points or greater. We included participants treated with allowed antipsychotics (amisulpride, haloperidol, olanzapine, risperidone, sulpiride, or tiapride, which are the most commonly prescribed antipsychotics in the participating countries), antidepressants, or other psychotropic drugs if they had received a stable dose for 6 weeks or longer before randomization.

used fluoxetine, paroxetine, tricyclic antidepressants, class 1 antiarrhythmics, or strong CYP2D6 inhibitors in the 6 weeks before randomization.

improvement (CGI-I) assessment, the Stroop word reading test, the UHDRS behavioural assessment, and the hospital anxiety and depression scale; secondary outcome measures were assessed at baseline (week 0) and at week 26. Our prespecified tertiary outcomes included changes in motor function, as measured by the UHDRS–TMS, and individual items within the mMS (gait and dysarthria). Our other prespecified tertiary outcomes included measures of cognitive function with the Stroop interference tests, the anxiety and depression subscales within the hospital anxiety and depression scale and the UHDRS behavioural

depression, fatigue, insomnia and so on.

			assessment sub scores, the UHDRS functional assessment, the UHDRS independence assessment, and the UHDRS functional capacity assessment.	
Lundin et al. 2010	The study included ambulatory patients aged 25 to 75 years who had been diagnosed on the basis of clinical features and detection of an expanded allele in the HTT gene.	Patients were excluded if they had clinically significant heart conditions or serum creatinine concentrations greater than 200 Kmol/L. Women of childbearing potential were excluded.	The primary objective of the study was to assess the effects of pridopidine on cognitive function. Secondary objective was to assess motor symptoms, affective symptoms, sleep quality, and safety and tolerability of the treatment. Outcome measures included the Unified Huntington's Disease Rating Scale (UHDRS) motor assessment, the Reitan Trail-Making Test A, the Leeds Sleep Evaluation Questionnaire, The Hospital Anxiety and Depression Scale	Measures of safety included the frequency and severity of reported adverse events (AEs) and changes in vital signs and electrocardiograms. Hematology and clinical laboratory tests were also performed. Tolerability was assessed by the number of patients

				(HADS), and the Clinical Global Impression of Change mental assessment scale.	who discontinued the study.

PRIDE-HD: Pridopidine Dose Evaluation in Huntington's Disease; HART: Huntington's Disease ACR16 Randomized Trial; MermaiHD: Multinational European Multicentre ACR16 Study in Huntington's Disease; HD: Huntington's disease; mMS: modified Motor Score; UHDRS-TMS: Unified Huntington's Disease Rating Scale-Total Motor Score; CAG: Cytosine-Adenine-Guanine; AE: adverse event; HTT: Huntingtin gene; HADS: Hospital Anxiety and Depression Scale; CGI-I: the Clinical Global Impression of Improvement; CGI-C: the Clinician Global Impression of Change.

Fig. S1 The sensitivity analysis of TMS

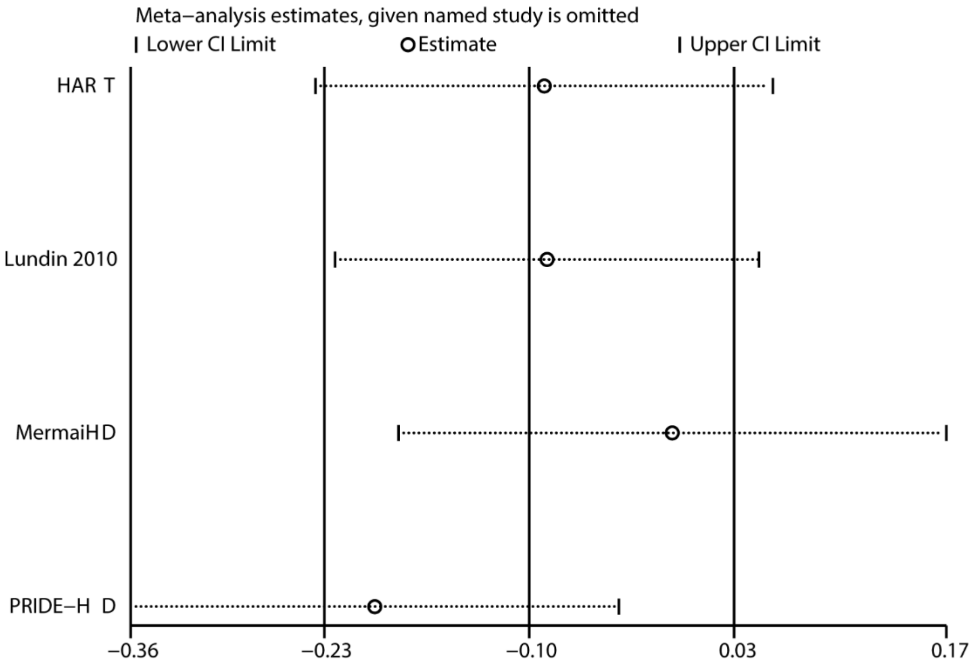

Supplement: Supplementary file 1 [file Data_Sheet_1.pdf]
